# Bemis v. Hayes

**Published:** 1882-06

**Authors:** M. P. Hatfield


### ARTICLE II.

BEMIS V. HAYES. By M. P. HATFIELD.

*Mrs. Emeline A. Bemis v. Dr. P. S. Hayes. Action in trespass, for* $10,000 *damages. Huszagh & Dunn for plaintiff. Heap & Mason for defendant.*

The history of the above case is so forcible an instance of the present legal status of the medical profession in Illinois, that it well deserves wider attention than it has yet obtained. The facts admitted by both parties to the suit are as follows:

Mrs. Bemis, a former patient of Dr. Hayes, applied to him for treatment July 15, 1879. She complained of frequent urination, headache, backache, bearing-down pains, and cessation of menses which she claimed had disappeared during her detention in the State Insane Asylum. A digital and speculum examination showed anteversion, subinvolution, and a left lateral laceration of the womb, for which Dr. Hayes used local and general treatment from July 15 to August 16—ten treatments in all—at the last of which he endeavored to replace the anteverted uterus by means of a rubber electrode used as a repositor. On the 17th, the next day, Mrs. Bemis rode some ten or twelve miles in a hack, and on the 19th suffered a miscarriage. On account of this Dr. Hayes was called on the 20th, removed a retained placenta, and after mutual recrimination, left her, telling her to summon another physician, if she needed further attention, as he would have nothing more to do with her, as she had willfully deceived him in reference to cohabitation with her husband.

After two years' unsuccessful efforts to compromise with Dr. Hayes, or to induce the district attorney to bring suit in her behalf, just before the case would have been barred by the statute of limitations, she at last found a lawyer who would undertake her case on the following counts, viz:

1st. Serious damages inflicted from a miscarriage brought about by Dr. Hayes' treatment.

2nd. Such treatment was the result of inexcusable ignorance on the part of Dr. Hayes, as Mrs. Bemis presented sufficient proof of pregnancy (fourth month) to have contraindicated the treatment adopted, even if she had not called attention to the cessation of her menses.

3d. Still further injury inflicted by Dr. Hayes' unskillful treatment at the time of the miscarriage, and especially by his refusal to further attend her when she was in great peril from his malpractice.

Defendant's answers to these charges were:

1st. No proof that the miscarriage was produced by treatment rather than by long ride taken on subsequent day.

2nd. The treatment adopted was entirely proper, provided plaintiff's statements in regard to cohabitation with her husband were accepted as true; and there were no proofs of pregnancy except those otherwise explainable by the uterine lesions.

3d. No injury was inflicted by leaving plaintiff, as Dr. Hayes was entirely justified, under the circumstances, in doing, after the removal of the placenta; and that injury, if any, was done by her failing to summon another physician until the 25th.

The points at issue in this case were, first, in regard to the relative truthfulness of Bemis and Hayes in regard to statements made as to symptoms, menstruation, cohabitation, and subsequent statements; and second, as to the danger incurred in leaving a woman, after a four months' miscarriage, temporarily without medical care, and as to the ability of ordinarily skillful physicians to diagnose the existence of pregnancy of three and one-half or four months, in a case like Mrs. Bemis'.

The first of these questions is the only one that an ordinary jury is competent to pass an opinion upon without expert testimony. Drs. W. H. Byford, D. T. Nelson, J. Adams Allen, R. B. Treat, W. L. Marr, A. J. Baxter, C. C. Davis, J. S. Wickersham and P. S. Hayes were summoned as experts, and with remarkable uniformity testified that the treatment given to Mrs. Bemis was entirely proper, if she were not pregnant. That it was extremely difficult, if not impossible (except, by W. L. Marr, by methods which he would not reveal to the court) to certainly diagnose pregnancy before quickening, and that the subjective symptoms in Mrs. Bemis' case were referable to other causes; and finally, that no serious injury was inflicted upon the plaintiff by leaving her as Dr. Hayes did.

Judge Williamson fairly charged the jury, that Mrs. Bemis' having paid, or failing to pay or promise to pay, in no wise debarred her from damages, if she had not received from the hands of Dr. Hayes such treatment as she ought from an ordinarily skillful physician; and, moreover, that damages, if any, ought to include recompense, not only for past and present injuries, but also for future disabilities arising from her present condition.

On the other hand, the present inquiry did not concern the general ability of the physician interested, but only the skill used at the times in question; but that if the treatment adopted was the result of untruthful statements made by the defendant, then she must be holden responsible for the results of such treatment.

With such instructions, a jury of medical men would have found a verdict for Dr. Hayes without leaving their seats; as it was, the jury disagreed. And why? Chiefly because, with the best of expert testimony, such a jury could not have the requisite knowledge for the just adjudication of such a case. A physician would hardly be willing to submit such a case to a jury of medical students; how much less to a jury who know absolutely nothing of medicine. A jury was originally made up of those who knew most about the case in question, but, according to the present system, one ignorant or bigoted juror can inflict a lifelong injury upon the most upright practitioner. Cases like these are worse than "eating porridge with the devil" for the young physician, for he has everything to lose, and nothing but a heavy lawyer's bill to gain, with even the most favorable result. Take the present case, for instance. Mrs. Bemis' lawyers probably are poorer than if she had gained her case, but she is no whit the worse off than when she began. Dr. Hayes' account stands about as follows: *credits*—the satisfaction of having done a kindly deed to a supposed friendless charity patient; *debits*—two years' persecution and annoyance, loss of time and professional reputation, and several hundred dollars legal expenses; for all of which the law at present gives him absolutely no recompense. Any malicious or designing patient, who can entrap a charitable physician into giving her his services may, on the flimsiest pretext, as in this case, bring an action in tort, or actually imprison the physician until the damages are paid in full.

And can there nothing be done to remedy this? Much, if there were concerted action on the part of the profession; but, as ever, everybody's business is nobody's, and the sufferer must look to his personal friends for assistance. Mrs. Bemis could not have brought suit for damages against the poorest saloon-keeper in Chicago, without finding that she had the whole liquor interest of the Northwest to contend against. It is the boast of Great Britain that the weakest one who bears the name of an English subject, is protected by the whole strength of England, and so it ought to be with our profession. Who will tell us how it can best be done?

---

EXTERNAL HÆMORRHOIDS.—Dr. Blaschko, of Berlin, recommends compresses soaked in a one per cent. solution of ergotin, to be applied hourly. Dr. Pasqua, of Florence, gives the following ointment as infallible.

Extr. of belladonna..................................gr. v.
Iodoform.......................................................
Acetate of lead... ..........................................aa gr. i.
Petroleum jelly............................................℥ i

Make into an ointment, to be applied three or four times a day.—*Druggists' Circular*.

PROLIFIC.—The 20th of December, 1881, I attended a lady of twenty years in her first confinement, who is next to the oldest of sixteen children (seven sons and nine daughters) ten of whom are still living. The father is now forty-one and the mother forty; both well and hearty. They were eighteen and nineteen respectively when married. All were single births and full term children. They live in New York State.—*A. H. Foster*.

